# Prevalence and predisposing factors of brachial plexus birth palsy in a regional hospital in Ghana: a five year retrospective study

**DOI:** 10.11604/pamj.2019.32.211.17914

**Published:** 2019-04-29

**Authors:** Cosmos Yarfi, Cephas Elekusi, Adjoa Nkrumah Banson, Seth Kwadjo Angmorterh, Nii Korley Kortei, Eric Kwasi Ofori

**Affiliations:** 1 Department of Physiotherapy and Rehabilitation Sciences, School of Allied Health Sciences, University of Health and Allied Sciences, Ho, Ghana; 2 Department of Medical Imaging, School of Allied Health Sciences, University of Health and Allied Sciences, Ho, Ghana; 3 Department of Nutrition and Dietetics, School of Allied Health Sciences, University of Health and Allied Sciences, Ho, Ghana

**Keywords:** Brachial plexus birth palsy, predisposing factors, erb’s palsy, Regional hospital, Ghana

## Abstract

**Introduction:**

Brachial plexus birth injury is one of the challenges associated with maternal delivery, with varying prevalence between countries. Brachial plexus birth injury poses negative health implications to children and also has socio-economic implications on families and the community as a whole. To treat brachial plexus birth injury, a multi-disciplinary treatment approach is recommended. Brachial plexus birth palsy (BPBP) is categorised into two-upper plexus injury (Erb's palsy) and lower plexus injury (Klumpke's palsy). These categories present with various degrees of injuries, with less severe injuries responding well to treatment and in most instances may resolve on their own, but serious and complicated injuries will require a multi-disciplinary treatment approach to treat and/or manage. Effective treatment and management depends on adequate knowledge of the disease condition. These include the risk factors and prevalence of brachial plexus birth palsy within a particular population at a specific period in time. The aim of this study was to determine the risk factors and the prevalence of a hospital based brachial plexus birth palsy within a five-year period (2013-2017).

**Methods:**

A five-year retrospective study design was used. The study involved selection of all clients' diagnosed with brachial plexus birth palsy, where their gender, birth weight, complications at birth, type of brachial plexus suffered, mothers' diabetes status, mother's age, birth attendant, side of affectation, presentation at birth and mode of delivery were recorded.

**Results:**

The prevalence rate of brachial plexus birth palsy was 14.7% out of a total of three hundred and twenty (320) cases reviewed over the study period in the Volta Regional Hospital. Erb's palsy was found to be the modal type of BPBP in this population (93.6%).

**Conclusion:**

There is the need to provide a nationwide education on the risk factors that predispose babies to brachial plexus birth palsy. There is also the need for frequent antenatal visit by pregnant women; this will help in the provision of best antenatal history, diagnostic investigation in determining the birth weight and safe mode of delivery.

## Introduction

Brachial plexus birth palsy (BPBP) is a neurological condition that results from nerve injury to the brachial plexus; C5-T1, which supply the upper extremities [1, 2]. Al-Qattan *et al.* [3] classified BPBP into four main groups; group one shows injury to C5-C6 and results in paralysis of the shoulder and biceps muscles. Group two affects C5-C7, and typically results in paralysis of the shoulder, biceps and the forearm extensors [4]. Group three indicates injury to C5-T1 and results in complete paralysis of the affected upper limb. Group four affects C5-T1 and results in complete paralysis of the affected upper limb with Horner's syndrome. Zafeiriou and Psychogiou [5] further indicated that group one shows the least severity of injury whereas group four indicates the most severity of injury. Upper plexus injury is an injury to C5, C6 and sometimes C7 nerve of the cervical spine and lower plexus injury are injury to C8, and T1 nerves of the thoracic spine [6, 7]. The management of BPBP has many physical, psychological, financial, social and emotional implications on families and the country as a whole [8]. The prevalence of BPBP varies among countries. According to Foad *et al.* [9], the prevalence of BPBP in the United States is 1.5 in 1000 live births; shoulder dystocia had 100 times greater risk, an exceptionally large baby (>4.5 kg) had a fourteen times greater risk, and forceps delivery had a nine times greater risk for having a child with brachial plexus birth palsy. Having a twin or multiple birth mates and delivery by cesarean section had a protective effect against the occurrence of neonatal brachial plexus palsy. A study conducted by Evans-Jones and colleagues [10], reported the prevalence in the United Kingdom to be 0.42 per 1000 live births and the associated risk factors for BPBP was found to be shoulder dystocia, high birth weight and assisted delivery, but a considerably lower risk in infants delivered by caesarean section.

The prevalence of children with BPBP in Nigeria over a ten year study period showed a persistent high prevalence, averaging 15.3% per year with associated problems such as birth asphyxia, humeral fracture, clavicular fracture and shoulder dislocation [4]. A study by Hamzat and colleagues [11] also reported the prevalence of BPBP in Accra, Ghana to be 27%, the results of the study further indicated that birth weight exceeding 4.0 kg, vertex presentation and vaginal delivery were the noticeable co-existing factors for BPBP in Accra. From the study done by Hamzat *et al*. [11] it was reported that only 55.2% of BPBP cases were referred for physiotherapy within one month after diagnosis and the treatment disposition for majority (88.1%) of the children were not documented and only 4.8% were formally discharged from physiotherapy. The prevalence of BPBP reported in Ghana focused on the Accra metropolis and there is no known data of BPBP in the Ho municipality, Volta region. The Ho municipality is a cosmopolitan urban city with health facilities serving the southern and central parts of the Volta region. BPBP is preventable when the risk factors and causes are known [12]. This work seeks to find out the prevalence and risk factors of BPBP in the Ho municipality of Ghana so as to advice policy makers on how to prevent the injury which will reduce the cost of treatment and increase productivity of the mothers. Managing of some children suffering from BPBP may need surgery and rehabilitation which poses financial issues to the family and loved ones [8]. Some children may have residual functional deficits and thereby affecting the way they function, as well psychological or emotional problems to the child [8]. This present study was undertaken to retrospectively investigate the prevalence of children who presented with BPBP and their predisposing factors in a regional hospital in Ghana. The clinical implication for this study is that there is not much information concerning the predisposing factors of BPBP in Ghana, apart from the study done by Hamzat *et al*. [11] that was done 10 years ago in Accra, an urban city in Ghana. Moreover the study by Hamzat *et al.* [11] did not provide information on the risk factors of BPBP in Ghana. As a result, there is paucity of information on the predisposing factors and the current prevalence of BPBP in a peri-urban city in Ghana. The aim of this study was to determine the prevalence and predisposing factors of BPBP over a five year period (January 2013-December 2017) at a regional hospital in Ghana.

## Methods

**Study site:** the study was conducted at the physiotherapy department of the Volta Regional Hospital (VRH), the main referral centre for the Volta region of Ghana. The hospital attends to over 177,281 inhabitants in the Ho municipality and its environs [13].

**Study design:** the study design employed was a retrospective quantitative study over a five-year period (January 2013 to December 2017).

**Ethical approval:** the study was approved by the Research Ethics Committees of the University of Health and Allied Sciences and the VRH with protocol identification number UHAS-REC/A.5 (62) 17-18.

**Inclusion and exclusion criterion:** all paediatric cases under 15 years who attended the physiotherapy clinic of the VRH over the study period were recruited. This age category is chosen because children within this age group are treated in the paediatric unit of the department. Paediatric cases above 15 years of age who reported for treatment over the study period were excluded. Also acute brachial plexus injuries not associated with delivery were also excluded.

**Procedure for data collection:** records of all newly diagnosed clients' were retrieved to identify all paediatric conditions. Sampling of all children diagnosed with birth brachial injuries were done, and this provided a means of recording their folder numbers for further investigation. The folder numbers obtained aided in the retrieval of individual folders. The study involved all children 15 years and below who attended the physiotherapy clinic of the VRH within the study period. The second stage of data collection included children that have been diagnosed with brachial plexus birth palsy within the study year period. Demographic data and clinical profile of children and their mothers were recorded. Information such as mother's history of birth, age at delivery, maternal diabetic status, parity status as well as the mothers' occupation was recorded. Other information retrieved included the diagnosis, side of affectation, birth weight, place of delivery, complication at birth, mothers' occupation, antenatal care history, type of BPBP, presentation at birth and others were retrieved from the client folders. The clients were grouped in two categories of Erb's and Klumpke's palsy. Those who had good wrist control were in Erb's classification while those with minimal to less wrist control but good shoulder control were classified as Klumpke's palsy. Those with no control of shoulders and wrist were classified as having total BPBP. Each participant was assigned a code to ensure anonymity. The data was kept under lock and key, accessible only by the researchers.

**Data analysis:** the data collected was entered into IBM SPSS version 20.0 analysed using descriptive statistics.

## Results

In all, the records of 47 participants who presented for BPBP were analysed. There were 28 (59.6%) female children with brachial plexus injuries, 17 (36.2%) were male children, and the gender for two (4.2%) of the cases were not documented. The average birth weight was 3.9 ± 0.5 kg. Table 1 presents the gender distribution for children diagnosed with BPBP in the five-year period with average birth weight and side of affectation. Table 2 shows the total number of paediatric conditions seen at the physiotherapy department of the Volta Regional Hospital during the study period. The weight category for children diagnosed with brachial plexus injuries was also recorded during the 5-year study period. Thirty-seven (37) of the children involved in the study had their birth weights recorded and their weights were categorised as shown in Table 2. The remaining 17.8% (n=8) of the children involved in the study had their birth weight not recorded. There was about half of brachial plexus injuries 48.7% (n=18) falling within the weight category (3.5 - 3.9 kg), 13.5% (n=5) falling within 3.0-3.4 kg and 24.3% (n=9) falling within the 4.5-5.0 kg weight category. Out of 320 paediatric cases reviewed during the study period, 47 (14.7%) of them were diagnosed with brachial plexus birth palsy. The rest of the paediatric cases constituted cerebral palsy (30.3%), musculoskeletal injuries (26.6%), acute flaccid paralysis (24%) and burns (4.4%). Brachial plexus birth palsy was the fourth (4^th^) leading cause for paediatric consultation and treatment at the physiotherapy department of the hospital.

**Table 1:** Gender distribution with their average birth weight and side of affectation

Gender	No of Babies	Percentage	Mean Birth Weight	Side of Affectation	
	n	%	Kg	Right	Left
Male	17	36.2	3.9±0.6	15	2
Female	28	59.6	3.1±0.4	19	7
Not indicated	2	4.2	3.8±0.2	2	-
**Total**	**47**	**100**		**39**	**9**

**Table 2:** Paediatric cases seen over the study period

Clinical diagnosis	n (%)
**Weight categories for BPBP (kg)**	
3.0-3.4	5(13.50)
3.5-3.9	18(48.7)
4.0-4.4	5(13.50)
4.5-5.0	9(24.30)
Paediatric cases	
BPBP	47(14.70)
Others	273(85.30)

The clinical profile of the babies diagnosed with BPBP indicated that, the vast majority 44 (93%) had cephalic presentation at birth, 1 (2.1%) had breech presentation and 2 (4.2%) of the cases were not documented. Majority of the children 44 (93.6%) were delivered through spontaneous/normal vaginal delivery, 2 (4.3%) through caesarean section. However, the mode of delivery of one baby was not documented. 44 (93.6%) of the cases were diagnosed with Erb's palsy whereas 3 (6.4%) had Klumpke's palsy. Shoulder dystocia and prolonged labour were the most common complications documented; 13 (27.7%) and 20 (42.5%) respectively. 2 (4.3%) of the children had fractures of the clavicle and humerus. Unfortunately, there were no records on 12 (5.5%) of the children. In Table 3, the ages of the mothers were compared to the average birth weight of their children and complications such as shoulder dystocia and prolonged labour. Out of a total of nineteen (19) of mothers' whose age were documented, only thirteen (13) of their children's birth weight were documented. Within the age categories of 15-24, 25-34 and above 34 years of mothers' age, 71%, 75% and 80% of children's birth weight respectively were documented. Mother's whose ages were above 34 years recorded the highest average birth weight (4.07 ± 0.60 kg) for their children together with shoulder dystocia, n=6 and prolonged labour n=6. The number of children born with shoulder dystocia was directly proportional to the mothers' age.

**Table 3:** Mothers’ age category versus average birth weight, shoulder dystocia and prolonged labour

Mothers’ age category	Average birth weight (Kg)	Shoulder dystocia (n)	Prolonged labour (n)
15- 24	3.90±0.57	1	2
25- 34	3.72±0.27	3	2
>34	4.07±0.60	6	6
**Total**		**10**	**10**

Data is presented in mean ± SD

## Discussion

The aim of this study was to determine the prevalence of brachial plexus birth palsy (BPBP) over a five year period (January 2013-December 2017) and the predisposing factors of BPBP at a regional hospital in Ghana. The clinical profiles of 320 paediatric cases, within the study period were reviewed. The prevalence of BPBP was 14.7%, which is much lower than the prevalence (27.2%) recorded in a tertiary hospital in urban Ghana [11]. The difference in the prevalence could be attributed to the geographical difference (urban vs. rural). Majority (59.6%) of the cases recorded were females, with 80% of the cases affecting the right arm. This result is consistent with the findings of studies conducted by Toopchizadeh and colleagues [14] in Tabriz, Iran. Hamzat *et al.* [11] also found out, right arm palsy to be more prevalent than left arm palsy. In the Ghanaian, context, the high prevalence of right arm palsy could pose social problems for children because the Ghanaian culture tends to place much emphasis on the importance of the right hand in performing actions such as greeting, eating, handshaking and preparing meals [11]. The implication therefore is that children with right hand palsy may be seen as outcasts within the Ghanaian societal setup.

Most (93.6%) of the study participants were cephalic spontaneous vaginal delivery with only two caesarean section (4.2%) and one having no documentation on the mode of delivery. About 95% of cephalic presentations were diagnosed with Erb's palsy, with 5% diagnosed with Klumpke's palsy; a breech presentation also resulted in Erb's palsy. These results confirm with a study conducted by Hale *et al.* [15], where they reported hyper abduction in breech presentation as a cause for Klumpke's palsy. For the current study, the high number of erb's palsy recorded for cephalic presentation will be due to the high birth weight of the children. Cephalic presentation with shoulder dystocia was recorded as the main mechanism putting a traction force on the upper brachial plexus [16]. The mechanism for Erb's palsy was reported to be due to, a traction force between the head and the shoulder, thereby putting a tension force on the nerve which might lead to tearing of some part or the entire upper brachial plexus [16]. This position supports the reason why majority of the children suffered from Erb's palsy in this study. According to Hehir and colleagues [16], cephalic presentation increase the risk for shoulder dystocia and causes a tension force on the upper brachial plexus, thereby leading to Erb's palsy in cases where the nerves are injured. The birth complications recorded in this study were shoulder dystocia (27.6%), prolonged labour (42.5%), clavicular fracture (2.1%) and humeral fracture (2.1%). These findings correspond to existing literature documenting shoulder dystocia and prolonged labour as the commonest complication for BPBP, with fractures recorded in some cases [17]. Similarly, Coroneos *et al.* [18] reported shoulder dystocia as the major cause for BPBP, but also found out that prolonged labour is the commonest risk factor resulting in BPBP. The recorded birth weight for the 47 BPBP participants used in this study were in the ranges of 3.0-5.0 kg with an average birth weight of 3.9 ± 0.5 kg. This finding shows that majority of the children's birth weight was within normal ranges (3.5-4.3 kg). This is consistent with existing literature which indicates that increase birth weight is a risk factor for BPBP [17].

The complications resulting in brachial plexus injury in this population is due to mothers not attending antenatal clinics, refusal to undergo caesarean section, delay in reporting to the hospital when labour sets in and lack of early referrals. Among the sampled population, majority of them suffered Erb's palsy (93.6%) with only 6.4% recording Klumpke's palsy. This shows that the number of children who suffered upper brachial injury was more than the lower plexus injuries. The clinical implication of this is that, clinicians and healthcare providers must be educated on the risk factors for brachial plexus injuries. Among the study population, only 19 mothers had their demographic details recorded. The average age and average birth weight of nulliparous mothers was 21 years and 3.8 kg respectively; whereas that for multiparous mothers was 34 years and 3.9 kg respectively. This shows that both nulliparous and multiparous mothers are at risk for delivering babies who may suffer birth injuries because the average birth weight is the same for both groups of women and therefore caregivers must provide the same level of care to prevent birth injuries. This study found out that age and parity status are risk free for brachial plexus injuries, since they are all at risk for the condition. This study contravenes other studies where mothers above 35 years and those below 16 years are considered as risk factors for brachial plexus injuries [19]. Birth weight above 4 kg was associated with an increased risk of shoulder dystocia and prolonged labour. Additionally, there was also an associated increased risk of shoulder dystocia, high birth weight (macrosomia) and prolonged labour in mothers above 34 years. This finding is consistent with the major risk factors reported by various studies [20, 21].

**Limitations:** this study was limited by the lack of complete and adequate maternal data. There was lack of documentation on birth weight for ten of the children and therefore conclusions about the study population cannot be representative.

## Conclusion

In conclusion, the results of this study showed a BPBP prevalence of 15% and most of the Erb's palsy cases had right arm affectation. The study also showed that majority of the babies had a birth weight >4.0 kg and this may accounts for the high number of shoulder dystocia cases. The presentations at birth were predominantly cephalic and this may pose an increased risk of upper plexus injuries among the macrosomic children. Majority of babies diagnosed with Erb's palsy were macrosomic with shoulder dystocia. **Recommendations:** the researchers recommend the need for appropriate documentation in the hospital, and the need for healthcare professionals to be mindful of the complications and the risk factors of BPBP so as to provide immediate and appropriate care.

### What is known about this topic

- Increase birth weight is a risk factor for BPBP;
- Birth weight above 4 kg is associated with an increased risk of shoulder dystocia and prolonged labour.

### What this study adds

- Increasing mothers' age was found to be directly related to the complications arising of the birth process such as prolonged labour and shoulder dystocia;
- Both nulliparous and multiparous mothers' are at risk for delivering babies who may suffer birth injuries because the average birth weight of children is the same for both groups of women;
- There is an associated increased risk of shoulder dystocia, high birth weight (macrosomia) and prolonged labour in mothers above 34 years.

## Competing interests

The authors declare no competing interests.
